# Knowledge, Attitude, and Practices about Polio Vaccination of the Guardian in Super High-risk Areas of Gadap Town, Karachi, Pakistan

**Published:** 2017-05

**Authors:** Amjad MIRANI, Kourosh HOLAKOUIE-NAIENI, Reza MAJDZADEH, Shahrzad NEMATOLLAHI, Saima BAIG

**Affiliations:** 1. International Campus, School of Public Health, Tehran University of Medical Sciences, Tehran, Iran; 2. Dept. of Epidemiology and Biostatistics, School of Public Health, Tehran University of Medical Sciences, Tehran, Iran; 3. Dept. of Media Studies Social Sciences, Sindh Madressatul Islam University, Karachi, Pakistan

## Dear Editor-in-Chief

Over 90% of Polio cases in Pakistan have been reported from four major transmission zones in Fata, Khyber-Pakhtunkhwa (K-P), Baluchistan, central Punjab and Sindh

Despite comprehensive governmental efforts since 1994 to eradicate polio (1), Pakistan is still facing substantial challenges such as inadequate program management, parental refusal and opposition of vaccination from the local groups (2). Media, religious leaders, community leaders and health care providers had vital role for dissemination of key messages about Polio.

Therefore, this study aimed to determine Knowledge, Attitude and Practice (KAP) of guardians about Polio vaccination and related factors in Super High-risk Areas of Gadap Town and put recommendation in form of a preventive action plan.

We assessed the KAP of guardians towards Polio vaccination. Totally, 554 guardians with children less than 5 yr of age were drawn from three Super High-risk Areas of Gadap Town in Karachi in 2016, using cluster sampling technique. A KAP questionnaire was administered. The results were further used to develop an action plan in order to increase KAP of guardians regarding Polio vaccination.

This cross-sectional descriptive study was approved by Ethical Committee of School of Public Health, Tehran University of Medical Sciences (TUMS), Tehran, Iran. Verbal informed consent prior to questionnaire completion was sought from the study subjects.

The mean age of study respondents was 34 (± 9.62) yr. Sixty percent of the guardians had private job, 40% of them had piped tank toilet, and 37% did not use treated water. Most of the guardians had knowledge about Polio as a health problem, clinical symptoms, a cause of permanent disability; while 58.16% were used to keep child`s vaccination card. Main sources of information about polio were healthcare providers, media, and local leaders. Totally, 86% of the guardians had positive attitude regarding polio vaccination including no fear of side effects, accessibility of health care facility, and promotion of local and religious leaders through education. 73.1% of guardians agreed that vaccination in form of EPI (i.e. Expanded Program on Immunization) could be an effective tool to protect children from Polio disease, while 91.2% agreed that proper hygiene & care could protect children from contracting Polio. In the current study, majority of the guardians had good knowledge about Polio disease. The guardians were informed about Polio mainly from media, health workers, and local community.

Our findings consistent with others showed that media, religious leaders, community leaders and health care providers have a vital role for dissemination of key messages about Polio (3, 4). We developed an action plan targeting guardians as well as community leaders. The action plan is aimed to change the behavioral communication, develop positive attitudes that promote and sustain behavior changes in individual, communities, and societies; and to maintain appropriate behavior of guardians about Polio vaccination program at super high-risk areas. The plan consisted of monthly educational programs on main results of the study such as improving the attitude regarding false beliefs, practice of proper waste-disposal, practice on hygiene and proper hand washing, practice of keeping vaccination card, and educational programs for local leaders (Table 1).

**Table 1:** Percentage distribution of practice of guardians about Polio vaccination

**Item**	**Yes**		**Do not know**		**Total**	
	n	%	n	%	n	%
Having their child vaccinated	494	89.2	60	10.9	554	100
Possessing vaccination card	323	58.3	231	41.7	554	100
Proper waste-disposal	520	93.9	34	6.1	554	100
Avoiding contact with infected person	380	68.6	174	31.4	554	100
Proper hand washing	543	98	11	1.9	554	100
